# Establishment of Novel Protein Interaction Assays between Sin3 and REST Using Surface Plasmon Resonance and Time-Resolved Fluorescence Energy Transfer

**DOI:** 10.3390/ijms22052323

**Published:** 2021-02-26

**Authors:** Masamitsu Harada, Jun Nagai, Riho Kurata, Xiaofeng Cui, Takayuki Isagawa, Hiroaki Semba, Yasuhiro Yoshida, Norihiko Takeda, Koji Maemura, Tomo Yonezawa

**Affiliations:** 1 Center for Therapeutic Innovation, Gene Research Center for Frontiers Life Sciences, Nagasaki University, Graduate School of Biomedical Sciences, 1-12-14 Sakamoto, Nagasaki 852-8523, Japan; vokuaoto@me.com (M.H.); JNAGAI@PARTNERS.ORG (J.N.); 2 Education and Research Center for Pharmaceutical Sciences, Osaka University of Pharmaceutical Sciences, 4-20-1 Nasahara, Takatsuki, Osaka 569-1094, Japan; kurata@gly.oups.ac.jp; 3 School of Chemistry, Chemical Engineering and Life Sciences, School of Materials and Engineering, Wuhan University of Technology, 122 Loushi Rd, Wuhan 430070, China; xfc.cui@gmail.com; 4 Data Science Center, Jichi Medical University, 3311-1 Yakushiji, Shimotsuke 329-0498, Japan; i-takayuki13@jichi.ac.jp; 5 Department of Cardiovascular Medicine, The Cardiovascular Institute, Nishiazabu 3-2-19, Minato-ku, Tokyo 106-0031, Japan; hiroaki_se@yahoo.co.jp; 6 Department of Immunology and Parasitology, University of Occupational and Environmental Health, 1-1 Iseigaoka, Yahatanishi-ku, Kitakyushu 807-8555, Japan; freude@med.uoeh-u.ac.jp; 7 Division of Cardiology and Metabolism, Center for Molecular Medicine, Jichi Medical University, 3311-1 Yakushiji, Shimotsuke 329-0498, Japan; ntakeda-tky@jichi.ac.jp; 8 Department of Cardiovascular Medicine, Graduate School of Biomedical Sciences, Nagasaki University Hospital, 1-7-1 Sakamoto, Nagasaki 852-8501, Japan; maemura@nagasaki-u.ac.jp

**Keywords:** REST, Sin3B, PAH1, SPR, TR-FRET

## Abstract

Repressor element-1 (RE-1) or neural restrictive silencer element (NRSE) bound with a zinc finger transcription repressor, RE-1 silencing transcription factor (REST, also known as neural restrictive silencer factor, NRSF) has been identified as a fundamental repressor element in many genes, including neuronal genes. Genes regulated by REST/NRSF regulate multifaceted neuronal phenotypes, and their defects in the machinery cause neuropathies, disorders of neuron activity), autism and so on. In REST repressions, the N-terminal repressor domain recruits Sin3B via its paired amphipathic helix 1 (PAH1) domain, which plays an important role as a scaffold for histone deacetylase 1 and 2. This machinery has a critical role in maintaining neuronal robustness. In this study, in order to establish protein–protein interaction assays mimicking a binding surface between Sin3B and REST, we selected important amino acids from structural information of the PAH1/REST complex and then tried to reconstitute it using recombinant short peptides derived from PAH1/REST. Initially, we validated whether biotinylated REST interacts with glutathione S-transferase (GST)-tagged PAH1 and whether another PAH1 peptide (PAH1-FLAG) competitively binds with biotinylated REST using surface plasmon resonance (SPR). We observed a direct interaction and competitive binding of two PAH1 peptides. Secondly, in order to establish a high-throughput and high-dynamic-range assay, we utilized an easily performed novel time-resolved fluorescence energy transfer (TR-FRET) assay, and closely monitored this interaction. Finally, we succeeded in establishing a novel high-quality TR-FRET assay and a novel interaction assay based on SPR.

## 1. Introduction

In 1992, two groups found repressor element-1 (RE-1) and bound it to a zinc finger transcription repressor in the *SCN2A*, *SCG10* and synapsin genes [1,2]. RE-1 silencing transcription factor (REST), which is also known as the neural restrictive silencer factor (NRSF) [3], was found to be an elemental repressor of transcription when binding to RE-1 [4] or neural restrictive silencer element (NRSE) [1] consisting of a 21-bp DNA element in many genes, including neuronal genes [3,4,5]. Genes regulated by REST/NRSF were reported to have functions in synaptic transmission [5,6], neurotransmitter signaling [7,8,9], ion channeling [4,10] etc. Ectopic expression of REST/NRSF or changes in the expression cellular pattern lead to symptoms of neuropathies such as medulloblastoma [11], glioblastoma [12], Huntington’s disease [13], neuropathic pain [14], Parkinson’s disease [15] and autism [16]. Thus, REST/NRSF plays a critical role in neuronal differentiation and functions and regulates other types of cells that do not express neuronal-specific genes. 

REST has two repressor domains at both N- and C-termini, respectively, and two co-repressor complexes [17,18]. The N-terminal repressor domain recruits mSin3 A and B [17], which are mammalian homologues of Yeast Sin3, and serve as a scaffold for histone deacetylase (HDAC) 1 and 2 [18]. Sin3B possesses four paired amphipathic helix domains (PAHs), PAH1–4 [19]. Previous structural analysis indicates that the PAH1 domain plays a crucial role in recruiting HDACs in the N-terminal repressor domain and its functions, because hydrophobic PAH1 is preferable for REST compared to the other major component co-repressor for REST (CoREST) [20]. While the C-terminal repressor domain recruits CoREST, G9a [20], a histone H3K9 methylase, or LSD1 [21], a histone H3K4 demethylase, it also recruits HDACs. 

In this study, in order to establish protein–protein interaction assays mimicking a binding surface between Sin3B and REST, we validated the interaction surface based on structural information about Sin3B and REST and then tried to establish it using recombinant short peptides generated by *Escherichia coli*. At first, we validated whether biotinylated REST interacted with GST-tagged PAH1 and whether another PAH1 peptide (PAH1-FLAG) competitively bound biotinylated REST using SPR. We could observe a direct interaction of these and competitive binding. Next, in order to establish a high-throughput and high-dynamic-range assay, we tried to establish a novel time-resolved fluorescence energy transfer (TR-FRET) assay easily and sensitively while monitoring this interaction. We succeeded in establishing a novel high-standard TR-FRET assay and a novel interaction assay based on SPR using biotinylated REST and GST-tagged PAH1.

## 2. Results

### 2.1. Binding Surface of Sin3B and REST, and Design of Interaction Assay

**Figure 1:**
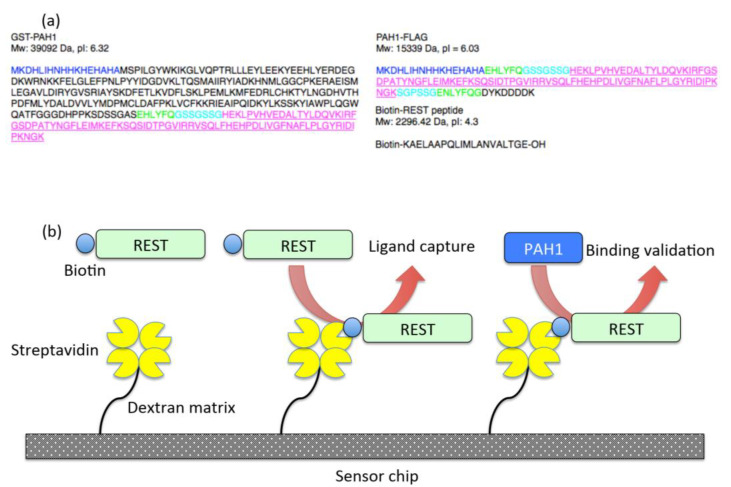
Binding surface of Sin3B and REST, and design of the interaction assay. (**a**) We designed recombinant GST-PAH1 (left), PAH1-FLAG (upper right) and biotinylated REST (lower right). Navy blue indicates histidine-tag-like sequences. Black indicates GST or FLAG tags except in biotinylated REST. Green indicates a TEV recognition site. Light blue indicates the flexible linker. Magenta indicates PAH1 or REST sequences consisting of their binding surface. Underlines indicate original peptides as previously reported [19]. (**b**) Schematic pictures indicate the SPR experiments in this study.

In order to validate a binding surface between Sin3B and REST, we utilized the structural information of PDB (2CZY) and designed longer peptides of binding surfaces with a tag and a protease recognition site as shown in Figure 1a. We asked the RIKEN Systems and Structural Biology Center (Yokohama, Japan) to generate recombinant peptides using *E*. *coli*. Finally, we obtained biotinylated REST, GST-PAH1 and PAH1-FLAG. In order to validate whether biotinylated REST binds two PAH1 peptides, we designed an SPR assay using a streptavidin (SA) sensor chip as shown in Figure 1b. Biotinylated REST should be bound as a ligand followed by the injection of PAH1 peptides as an analyte.

### 2.2. Protein–Protein Interaction between REST and PAH1 Peptides

**Figure 2:**
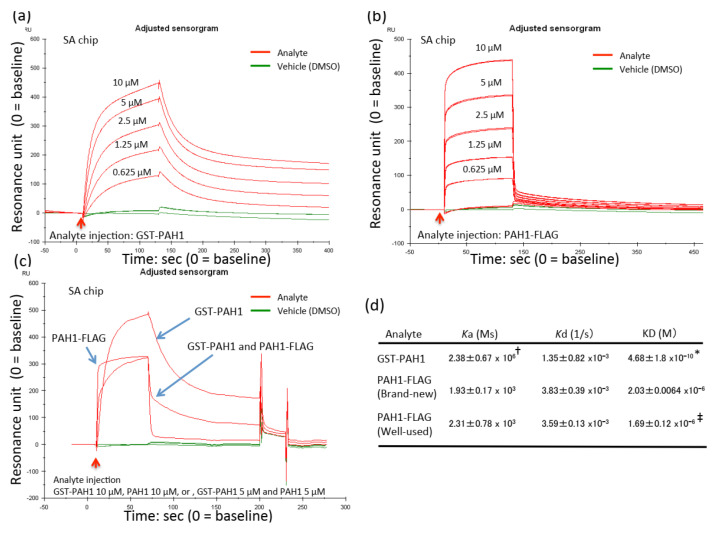
Protein–protein interaction between REST and PAH1 peptides. Sensorgram of various concentration of GST-PAH1 (**a**) or PAH1-FLAG (**b**) binds biotinylated REST bound with a streptavidin (SA) chip. Red arrows indicate the injection of each analyte, and the red lines track their resonance unit over time. Green indicates injection of vehicle (DMSO). These experiments are based on three series of experimental results with various concentrations of each analyte binding biotinylated REST as determined by SPR. (**c**) The results indicate 10 µM GST-PAH1, 10 µM PAH1-FLAG or 5 µM GST-PAH1 and PAH1-FLAG bound biotinylated REST bound with an SA chip. Red arrows indicate the injection of each analyte, and the red lines track their resonance unit over time. Green indicates the injection of vehicle (DMSO). (**d**) The summarized table indicates the association rate (*Ka*), dissociation rate (*Kd*) and dissociation constant (KD) of each analyte to biotinylated REST. GST-PAH1 and PAH1-FLAG (brand new) indicates measurements performed by a brand-new sensor chip immobilized with biotinylated REST. “Well-used” indicates experiments using the sensor chip after around 100 regenerations. The data are expressed as means ± SEMs (*n* = 3). * indicates they were considered to be statistically significant if their *p* values were 0.00005 > *p* between GST-PAH1 and PAH1-FLAG. † indicates a statistical trend between GST-PAH1 and PAH1-FLAG. ‡ indicates a statistical trend between brand-new and well-used. The statistical trend was considered to be statistically significant if their *p* values were 1 > *p* > 0.05.

In preliminary experiments, we succeeded in capturing biotinylated REST on the SA sensor chip (Supplementary Figure S1a,b). We observed a transient change of around 1500 resonance units (RU) and basal RU level (Supplementary Figure S1a). However, without ligand capturing, we could not observe the basal RU, although bulk effects caused around 200 RU (Supplementary Figure S1b). After ligand capturing, we obtained robust association and subsequent dissociation between biotinylated REST and GST-PAH1 (Supplementary Figure S1c), and to a lesser extent a change of RU was observed, probably caused by the bulk effect (Supplementary Figure S1d). We also determined an optimal wash condition using 10 mM glycine hydrochloride for the regeneration of the sensor chip, and then could perform the interaction assay over one hundred times (data not shown, Figure 2).

GST-PAH1 (Figure 2a) or PAH1-FLAG (Figure 2b) bound biotinylated REST in a concentration-dependent manner (Figure 2a). The concentrations were from 0.625 to 10 µM. We also performed at least three independent identical experiments, and then calculated *Ka*, *Kd* and KD (Figure 2d). The values with GST-PAH1 were 2.38 ± 0.67 × 10^6^, 1.35 ± 0.82 × 10^−3^ and 3.79 ± 1.8 × 10^−10^, respectively (Figure 2d, upper). Those with PAH1-FLAG were 1.93 ± 0.17 × 10^3^, 3.83 ± 0.39 × 10^−3^ and 2.03 ± 0.0064 × 10^−6^, respectively (Figure 2d, middle). After 100 regenerations, we observed the binding of PAH1-FLAG to sensor chip bound with biotinylated REST showing the values of 2.31 ± 0.78 × 10^3^, 3.59 ± 0.13 × 10^−3^ and 1.69 ± 0.12 × 10^−6^, respectively (Figure 2d, lower). Our statistical analysis between GST-PAH1 and PAH1-FLAG indicates that the association rate of GST-PAH1 was not significant but had a slower trend than PAH1-FLAG, resulting in a significantly increased affinity to REST (Figure 2d, upper). We also made a statistical comparison between a brand new and a well-used sensor chip bound with REST. The affinity of the well-used chip was not significantly different, but had a trend of lower affinity when compared to the brand new one; we could observe a substantial interaction in both chips (Figure 2d, lower).

In order to determine whether another PAH1 peptide, PAH1-FLAG, competitively binds biotinylated REST in the presence of GST-PAH1, we injected 10 µM GST-PAH1, 10 µM PAH1-FLAG or 5 µM GST-PAH1 and 5 µM PAH1-FLAG into the flow cell capturing biotinylated REST, all of which could change the sensorgram, even though their rates of association/dissociation were different (Figure 2c). Both rates of association and dissociation in PAH1-FLAG occurred far more quickly than in GST-PAH1 (Figure 2c). As a result, the equal molarity of both PAH1 peptides changed the sensorgram-like intermediate form (Figure 2c). The association rate was faster than GST-PAH1, while the dissociation rate was slower than PAH1-FLAG. The results indicate that PAH1-FLAG shares the same binding surface as GST-PAH1.

### 2.3. Establishment of Time-Resolved Fluorescence Energy Transfer Assay Mimicking Protein–Protein Interaction between PAH1 and REST

**Figure 3:**
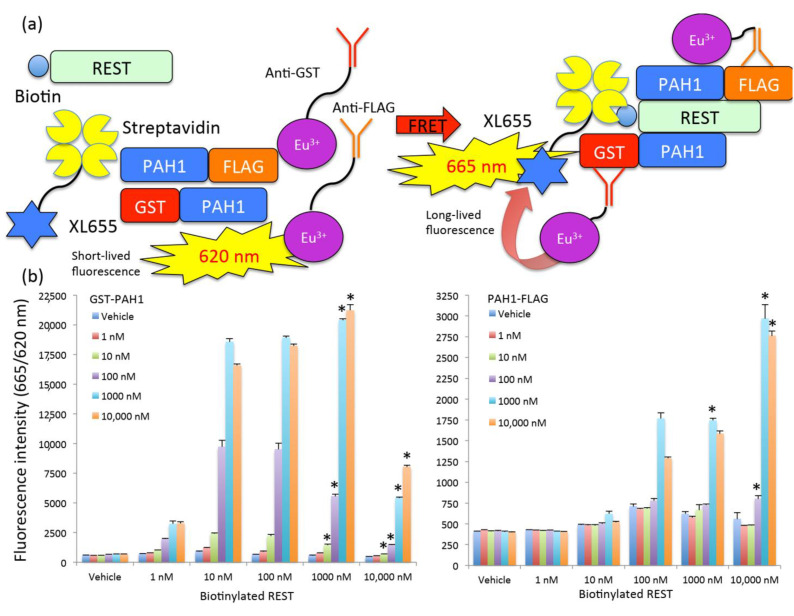
Establishment of time-resolved fluorescence energy transfer assay mimicking protein–protein interaction between PAH1 and REST. (**a**) Schematic figure indicates TR-FRET using GST-PAH1, PAH1-FLAG, biotinylated REST, antibodies-Eu^3+^ and XL665. (**b**) The results of TR-FRET using various concentration of GST-PAH1 (left) or PAH1-FLAG (right) with various concentrations of biotinylated REST. The data are expressed as means ± SEMs (*n* = 4). * considered to be statistically significant if *p* < 0.00005.

Finally, in order to establish a high-throughput and high-dynamic-range assay mimicking the binding surface between PAH1 and REST, we tried to apply the TR-FRET method for monitoring the interaction as shown in Figure 3a. We chose Europium cryptate (Eu^3+^)-conjugated antibodies against GST or FLAG as a donor and SA-conjugated XL665 (a phycobiliprotein pigment derived from red algae [22]) as an acceptor, for TR-FRET. If GST-PAH1 or PAH1-FLAG binds biotinylated REST and then transfers Eu^3+^’s energy to XL665, it results in a change of the wavelength from 620 nm (a short-lived fluorescence derived from Eu^3+^ alone) to 665 nm (a long-lived fluorescence converted by FRET) (Figure 3a). We tried to determine optimal concentrations of GST-PAH1 or PAH1-FLAG and biotinylated REST in the presence of anti-GST or anti-FLAG-Eu^3+^ and SA-XL655 for detecting substantial signals of 665 nm. We prepared a serial dilution of GST-PAH1 or PAH1-FLAG and biotinylated REST ranging from 1 to 10,000 nM (Figure 3b) and then determined the 665 nm fluorescence normalized to 620 nm. We obtained substantial signals between GST-PAH1 and biotinylated REST (Figure 3b, left), while between PAH1-FLAG and biotinylated REST we observed fewer signals only at high reagent concentrations (Figure 3b, right). We also checked whether GST-PAH1-anti-GST-Eu^3+^ or FLAG-PAH1-anti-FLAG-Eu^3+^ complexes caused any FRET with XL665 (Supplementary Figure S1e), and could not observe any change in wells with these alone. We assigned thresholds for high-throughput and high-standard assay as signal-to-background ratio (S/B) and Z’ factors of more than 5 and 0.8, respectively. We narrowed down optimal conditions to GST-PAH1 at 10, 100, 1000 or 10,000 nM in the presence of biotinylated REST at 1000 or 10,000 nM and that PAH1-FLAG and biotinylated REST at 10,000 nM. The S/B, signal-to-noise ratio (S/N), Z’ factor and coefficient of variation (CV) are shown in the Supplementary Table S1. We also assigned stringent statistical significance and then identified similar results by the S/B and Z’ factor (Figure 3b). To exclude the possibility that Eu^3+^ antibodies directly bound XL665 causing FRET, we also determined the fluorescence level in wells including anti-GST-Eu^3+^ or anti-FLAG-Eu^3+^ and XL665. We did not detect any significant changes in the wells, including anti-GST-Eu^3+^ or anti-FLAG-Eu^3+^ and XL665. The results were 772 ± 9.1 and 436 ± 9.1 665/620 nm, respectively (*n* = 6). Although we observed very good dynamic range data at 1 h after preparing reaction mixtures, we obtained similar high-dynamic range data even at 18 h after that (data not shown). From 10 to 1000 nM in the concentration of biotinylated REST, 1000 or 10,000 nM GST-PAH1 induced maximal signals (Figure 3b, left, light blue and orange). The dynamic range of the assay was 31-fold, whereas only 10,000 nM PAH1-FLAG was required for the same amount of biotinylated REST to provide a maximal response and the dynamic range was 7-fold (Figure 3b, right, orange). We ultimately succeeded in establishing a high-standard assay, especially using GST-PAH1 and biotinylated REST.

## 3. Discussion

In this study, we for the first time established novel protein–protein assays using PAH1 and REST peptides derived from whole Sin3B and REST molecules. Professor Nishimura’s group previously identified the structure of the binding surface between PAH1 and REST [19]. We posit that PAH1 and REST peptides, consisting of the binding surface, are far more elongated than peptides as previously reported [19]. We observed that elongated and tagged PAH1 peptides bound elongated REST peptides captured on an SA sensor chip using an SPR assay. Additionally, each PAH1 peptide competitively bound REST peptides, suggesting the two peptides share the same REST binding site. Indeed, even in TR-FRET we detected FRET caused by the protein–protein interaction of PAH1-REST peptides. To our knowledge, it was the first demonstration by SPR to analyze interactions between a PAH1 domain and a REST binding surface. In a previous report, Xie et al. demonstrated that PAH3 derived from Sin3A, which consists of 71 amino acid residues, has homology and shares important residues with PAH1, binds 91 amino acid residues, the C-terminal Sin3 interaction domain (SID) of the 30-kDa Sin3-assocated protein (SAP30) by SPR [23]. The values of *Ka*, *Kd* and KD were 1.07 ± 0.01 × 10^5^, 9.82 ± 0.33 × 10^−4^ and 9.2 ± 0. 4 × 10^−9^, respectively [23]. Our results indicate Ka, Kd and KD between PAH1 and REST peptides are reasonable values because we obtained similar or much higher affinity than those between PAH3 and SAP30 SID. Our results also indicate that the affinity of GST-PAH1 is significantly greater than that of PAH1-FLAG. GST tag protein may influence the affinity. We succeeded in detecting long-lived 665 nm fluorescence from XL665 converted by Eu^3+^ using both GST-PAH1 and PAH1-FLAG, but it was in a remarkably higher dynamic range and accuracy only when GST-PAH1 was used. It would be more efficient to transfer the donor energy into the acceptor because of the position of the tag at the C-terminus, the different affinity among antibodies or some other effect. However, it is unlikely that free Eu^3+^ cryptate reacts to free XL665. Finally, we established an assay with high dynamic range (around 30-fold), high accuracy (a CV value less than 5%) and a Z’ factor of around 0.9 to monitor the protein–protein interaction between PAH1 and REST. This would be suitable for performing high-throughput screening (HTS) to identify any inhibitors of the PAH1–REST interaction. After HTS, we can utilize SPR assay to validate the inhibition of compounds in detail. Thus, these novel interaction assays are very useful to develop drug discovery targeting Sin3–REST interaction. With respect to the throughput of each assay, if the TR-FRET is required for 2 h per assay, a single assay bears 320 samples in 384-plate format. During the same time, SPR can perform around 10 samples.

The compounds, which inhibit binding of Sin3 to REST, may affect not only diseases involving neuronal activities, but also affect pluripotent cells and their differentiation. Indeed, knockdown of Sin3A or B in P19 pluripotent cells leads to the enhancement of differentiation and neuronal phenotypes, resulting in electrophysiologically active neurons although the effects of Sin3A are greater than those of Sin3B [24]. Additionally, in breast cancer metastasis Sin3A and B have opposite effects [25]. Knockdown of Sin3B inhibits metastasis, while knockdown of Sin3A accelerates metastasis [25]. This novel high-dynamic range and high-accuracy assay can be utilized in performing diagnostics for neuropathies such as medulloblastoma [11], glioblastoma [12], Huntington’s disease [13], neuropathic pain [14], Parkinson’s disease [15], autism [16] and tumors [25].

In conclusion, we established two novel protein–-protein interaction assays based on SPR and TR-FRET. In the near future, we will perform HTS using TR-FRET assay to identify inhibitors of this interaction and then validate how this inhibits the interaction by SPR assay. This can be utilized in diagnostics against neuropathies and cancer metastasis and in regenerative medicine by engineering pluripotent stem cells.

## 4. Materials and Methods

### 4.1. Materials

Streptavidin (SA) sensor chip, HBS+ buffer and 50 mM NaOH were obtained from GE Healthcare, Uppsala, Sweden. Anti-GST-Eu^3+^ cryptate (#61GSTKLA), Anti-FLAG-Eu^3+^ cryptate (#61FG2XLA) and SA-XL665 (610SAXLA) were obtained from Cisbio International Laboratories, Gif sur Yvette, France). Tris-HCl, NaCl, MgCl_2_, BSA, KF and DMSO were obtained from Sigma-Aldrich, St. Louis, MO, USA. GST-PAH1, PAH-FLAG and biotinylated REST peptides were kindly gifted from Prof. Shirouzu, Prof. Niino and Dr. Ihara (RIKEN Systems and Structural Biology Center, Yokohama, Japan). The sequences of peptides are shown in Figure 1a.

### 4.2. Selection and Generation of PAH1 and REST Peptides

We chose the amino acid sequences of PAH1 and REST from information based on the crystal structure of a PAH1/REST complex (PDB: 2CZY) [19]. We then asked the scientists with the RIKEN institute to produce recombinant PAH1 and REST peptides. They generated recombinant peptides such as GST-PAH1, PAH1-FLAG and biotinylated REST using *E*. *coli*. We used these peptides in this study.

### 4.3. Surface Plasmon Resonance

We performed the SA-capturing protocol following Biacore T200 instructions. Biotinylated REST (10 µM) as ligand was injected into SA-tagged dextran on a sensor chip at 15 µL/min flow rate for 100 s. After the ligand captured the biotinylated REST, an analyte injection was done at 30 µL/min flow speed for 60 s in the association phase. The dissociation phase was then started without an analyte at the same flow rate for 2 min. Eventually, the 50 mM NaOH or 10 mM Gly-HCl (pH 2.0) injection washed away the ligand and/or analyte. We also performed a kinetics protocol using various concentrations of GST-PAH1 ranging from 0.625 to 10 µM in at least three independent experiments and then calculated the KD values of the PAH1–REST interaction. All measurements and analyses were done using Biacore T200 and its analysis Wizard software (GE Healthcare, Uppsala, Sweden).

### 4.4. TR-FRET

TR-FRET reagents were prepared by diluting the anti-GST Eu3+ cryptate or anti-FLAG Eu3+ cryptate and SA-XL665 in 50 mM Tris-HCl (pH 7.6) buffer containing 15 mM NaCl, 140 mM KCl, 0.5 mM MgCl_2_, 0.1% BSA and 0.8 M KF with and without biotinylated REST and each PAH1 peptide. The reaction mix was 20 µL in 384-well format according to the manufacturer’s instructions. The fluoride ion avoids interference with fluorescence properties by protecting free coordination sites between conjugate “cage” and europium ions. One hour or more after preparation, we measured the intensities at 665 and 620 nm wavelength using PHERAstar FS (BMG biotech, Offenburg, Germany).

### 4.5. Statistics

Values are expressed as the mean ± standard error of the mean (SEM) from indicated replicate samples in each experimental group and the experiments were replicated to ensure consistency. The statistical significance was determined using the Student’s *t*-test. Values marked as “*” were considered to be statistically significant if their *p* values were <0.00005. Signal-to-background ratio (S/B), signal-to-noise ratio (S/N) and Z’-factor were calculated by the formula described in our previous report [26]. Additionally, the general criterion sufficient to perform HTS was validated in our previous report [27,28,29]. In the SPR experiments, a statistical trend was considered to be statistically significant if its *p*-value was 1 > *p* > 0.05.

## Data Availability

The data that support the findings of this study are available from the corresponding author upon reasonable request.

## Supplementary Materials

The following are available online at https://www.mdpi.com/1422-0067/22/5/2323/s1.

## References

[1] Kraner S.D., Chong J.A., Tsay H.J., Mandel G. (1992). Silencing the type II sodium channel gene: A model for neural-specific gene regulation. Neuron.

[2] Mori N., Schoenherr C.J., Vandenbergh D.J., Anderson D.J. (1992). A common silencer element in the SCG10 and type II Na+ channel genes binds a factor present in nonneuronal cells but not in neuronal cells. Neuron.

[3] Schoenherr C.J., Anderson D.J. (1995). The neuron-restrictive silencer factor (NRSF): A coordinate repressor of multiple neuron-specific genes. Science.

[4] Chong J.A., Tapia-Ramirez J., Kim S., Toledo-Aral J.J., Zheng Y., Boutros M.C., Altshuller Y.M., Frohman M.A., Kraner S.D., Mandel G. (1995). REST: A mammalian silencer protein that restricts sodium channel gene expression to neurons. Cell.

[5] Bruce A.W., Donaldson I.J., Wood I.C., Yerbury S.A., Sadowski M.I., Chapman M., Göttgens B., Buckley N.J. (2004). Genome-wide Analysis of Repressor Element 1 Silencing Transcription Factor/Neuron-Restrictive Silencing Factor (REST/NRSF) Target Genes. Proc. Natl. Acad. Sci. USA.

[6] Schoch S., Cibelli G., Thiel G. (1996). Neuron-specific Gene Expression of Synapsin I. J. Biol. Chem..

[7] Wood I.C., Roopra A., Buckley N.J. (1996). Neural specific expression of the m4 muscarinic acetylcholine receptor gene is mediated by a RE1/NRSE-type silencing element. J. Biol. Chem..

[8] Bessis A., Champtiaux N., Chatelin L., Changeux J.-P. (1997). The neuron-restrictive silencer element: A dual enhancer/silencer crucial for patterned expression of a nicotinic receptor gene in the brain. Proc. Natl. Acad. Sci. USA.

[9] Bai G., Norton D.D., Prenger M.S., Kusiak J.W. (1998). Single-stranded DNA-binding Proteins and Neuron-restrictive Silencer Factor Participate in Cell-specific Transcriptional Control of the NMDAR1 Gene. J. Biol. Chem..

[10] Yeo M., Berglund K., Augustine G., Liedtke W. (2009). Novel repression of Kcc2 transcription by REST-RE-1 controls developmental switch in neuronal chloride. J. Neurosci..

[11] Lawinger P., Venugopal R., Guo Z.-S., Immaneni A., Sengupta D., Lu W., Rastelli L., Carneiro A.M.D., Levin V., Fuller G.N. (2000). The neuronal repressor REST/NRSF is an essential regulator in medulloblastoma cells. Nat. Med..

[12] Kamal M.M., Sathyan P., Singh S.K., Zinn P.O., Marisetty A.L., Liang S., Gumin J., El-Mesallamy H.O., Suki D., Colman H. (2012). REST Regulates Oncogenic Properties of Glioblastoma Stem Cells. Stem Cells.

[13] Zuccato C., Tartari M., Crotti A., Goffredo D., Valenza M., Conti L., Cataudella T., Leavitt B.R., Hayden M.R., Timmusk T. (2003). Huntingtin interacts with REST/NRSF to modulate the transcription of NRSE-controlled neuronal genes. Nat. Genet..

[14] Willis D.E., Wang M., Brown E., Fones L., Cave J.W. (2016). Selective repression of gene expression in neuropathic pain by the neuron-restrictive silencing factor/repressor element-1 silencing transcription (NRSF/REST). Neurosci. Lett..

[15] Suo H., Wang P., Tong J., Cai L., Liu J., Huang D., Huang L., Wang Z., Huang Y., Xu J. (2015). NRSF Is an Essential Mediator for the Neuroprotection of Trichostatin A in the MPTP Mouse Model of Park-inson’s Disease. Neuropharmacology.

[16] Katayama Y., Nishiyama M., Shoji H., Ohkawa Y., Kawamura A., Sato T., Suyama M., Takumi T., Miyakawa T., Nakayama K.I. (2016). CHD8 haploinsufficiency results in autistic-like phenotypes in mice. Nat. Cell Biol..

[17] Huang Y., Myers S.J., Dingledine R. (1999). Transcriptional repression by REST: Recruitment of Sin3A and histone deacetylase to neuronal genes. Nat. Neurosci..

[18] Andrés M.E., Burger C., Peral-Rubio M.J., Battaglioli E., Anderson M.E., Grimes J., Dallman J., Ballas N., Mandel G. (1999). CoREST: A functional corepressor required for regulation of neural-specific gene expression. Proc. Natl. Acad. Sci. USA.

[19] Nomura M., Uda-Tochio H., Murai K., Mori N., Nishimura Y. (2005). The Neural Repressor NRSF/REST Binds the PAH1 Domain of the Sin3 Corepressor by Using its Distinct Short Hydrophobic Helix. J. Mol. Biol..

[20] Roopra A., Qazi R., Schoenike B., Daley T.J., Morrison J.F. (2004). Localized Domains of G9a-Mediated Histone Methylation are Required for Silencing of Neuronal Genes. Mol. Cell.

[21] Shi Y., Lan F., Matson C., Mulligan P., Whetstine J.R., Cole P.A., Casero R.A., Shi Y. (2004). Histone Demethylation Mediated by the Nuclear Amine Oxidase Homolog LSD1. Cell.

[22] Mathis G., Buschow J.K.H., Cahn R.W., Flemings M.C., Ilschner B., Kramer E.J., Mahajan S., Veyssiere P. (2001). Bioassays: Luminescent Materials in Encyclopedia of Materials: Science and Technology.

[23] Xie T., He Y., Korkeamaki H., Zhang Y., Imhoff R., Lohi O., Radhakrishnan I. (2011). Structure of the 30-kDa Sin3-associated Protein (SAP30) in Complex with the Mammalian Sin3A Corepressor and Its Role in Nucleic Acid Binding. J. Biol. Chem..

[24] Halder D., Lee C.-H., Hyun J.Y., Chang G.-E., Cheong E., Shin I. (2017). Suppression of Sin3A Activity Promotes Differentiation of Pluripotent Cells Into Functional Neurons. Sci. Rep..

[25] Lewis M.J., Liu J., Libby E.F., Lee M., Crawford N.P., Hurst D.R. (2016). SIN3A and SIN3B differentially regulate breast cancer metastasis. Oncotarget.

[26] Kurata R., Kumagai A., Cui X., Harada M., Nagai J., Yoshida Y., Ozaki K.-I., Tanaka Y., Yonezawa T. (2018). Establishment of Novel Reporter Cells Stably Maintaining Transcription Factor-driven Human Secreted Alkaline Phosphatase Expression. Curr. Pharm. Biotechnol..

[27] Zhang J.-H., Chung T.D.Y., Oldenburg K.R. (1999). A Simple Statistical Parameter for Use in Evaluation and Validation of High Throughput Screening Assays. J. Biomol. Screen..

[28] Kurata R., Shimizu K., Cui X., Harada M., Isagawa T., Semba H., Ishihara J., Yamada K., Nagai J., Yoshida Y. (2020). Novel Reporter System Monitoring IL-18 Specific Signaling Can Be Applied to High-Throughput Screening. Mar. Drugs.

[29] Harada M., Nagai J., Kurata R., Shimizu K., Cui X., Isagawa T., Semba H., Ishihara J., Yoshida Y., Takeda N. (2020). Establishment of Novel High-Standard Chemiluminescent Assay for NTPase in Two Protozoans and Its High-Throughput Screening. Mar. Drugs.

