# Supplementary material for: Establishment of Novel Protein Interaction Assays between Sin3 and REST Using Surface Plasmon Resonance and Time-Resolved Fluorescence Energy Transfer
Source: Int J Mol Sci. 2021 Feb 26;22(5):2323. doi: 10.3390/ijms22052323 (PMC7956749; doi:10.3390/ijms22052323)
Supplement: Supplementary file 1 [file ijms-22-02323-s001.pdf]

Supplementary Figure S1

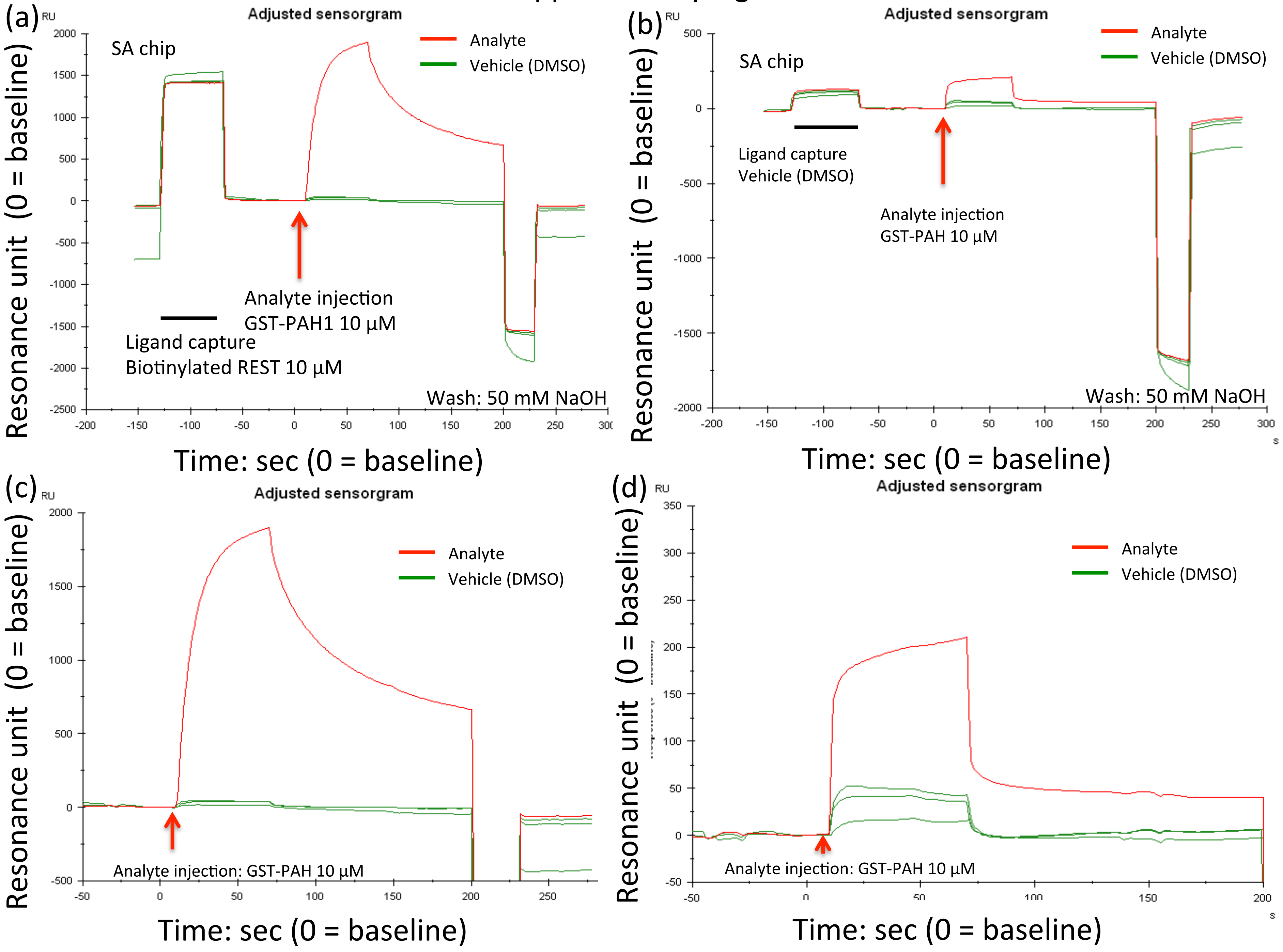

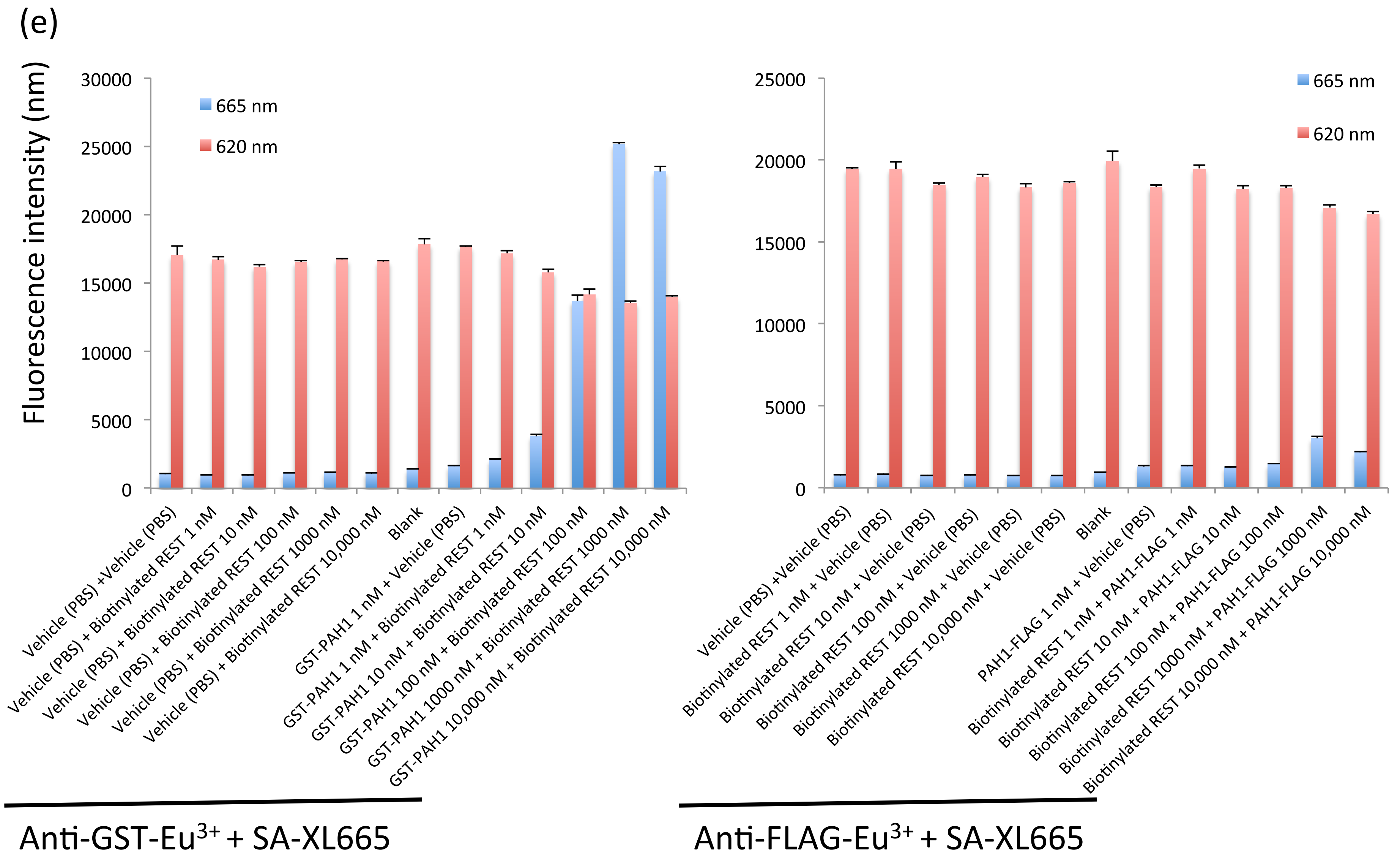
(e)
Fluorescence intensity (nm)
30000
25000
20000
15000
10000
5000
0
665 nm
620 nm
Vehicle (PBS) +Vehicle (PBS)
Vehicle (PBS) + Biotinylated REST 1 nM
Vehicle (PBS) + Biotinylated REST 10 nM
Vehicle (PBS) + Biotinylated REST 100 nM
Vehicle (PBS) + Biotinylated REST 1000 nM
Vehicle (PBS) + Biotinylated REST 10,000 nM
Blank
GST-PAH1 1 nM + Vehicle (PBS)
GST-PAH1 1 nM + Biotinylated REST 1 nM
GST-PAH1 10 nM + Biotinylated REST 10 nM
GST-PAH1 100 nM + Biotinylated REST 100 nM
GST-PAH1 1000 nM + Biotinylated REST 1000 nM
GST-PAH1 10,000 nM + Biotinylated REST 10,000 nM
Anti-GST-Eu^{3+} + SA-XL665
25000
20000
15000
10000
5000
0
665 nm
620 nm
Vehicle (PBS) +Vehicle (PBS)
Biotinylated REST 1 nM + Vehicle (PBS)
Biotinylated REST 10 nM + Vehicle (PBS)
Biotinylated REST 100 nM + Vehicle (PBS)
Biotinylated REST 1000 nM + Vehicle (PBS)
Biotinylated REST 10,000 nM + Vehicle (PBS)
Blank
PAH1-FLAG 1 nM + Vehicle (PBS)
Biotinylated REST 1 nM + PAH1-FLAG 1 nM
Biotinylated REST 10 nM + PAH1-FLAG 10 nM
Biotinylated REST 100 nM + PAH1-FLAG 100 nM
Biotinylated REST 1000 nM + PAH1-FLAG 1000 nM
Biotinylated REST 10,000 nM + PAH1-FLAG 10,000 nM
Anti-FLAG-Eu^{3+} + SA-XL665

Legends for Supplemenatry Figure S1

Sensor-gram of GST-PAH1 with (a) or without (b) capturing bitinylated REST bound with SA chip. (c) and (d) indicates enlarged sensor-grams of (a) and (b), respectively. Red indicates injection of each analyte. Green indicates injection of vehicle (DMSO).

(e) The results of TR-FRET at 665 and 620 nm using various concentration of GST-PAH1or PAH1-FLAG with various concentration of biotinylated REST in the presence of anti-GST-Eu3+ (left) or anti-FLAG-Eu3+ (right) with SA-XL665. The data are expressed as means ± SEM (n= 4).

Supplementary table S1

Qualities of independent experiments

REST	PAH1	S/B	S/N	Z'-factor	CV	Number of sample
1000 nM	GST-10 nM	26.6	469.9	0.91	2.8%	n=4
	GST-100 nM	27.0	478.7	0.94	1.7%	n=4
	GST-1000 nM	29.2	517.8	0.96	1.2%	n=4
	GST-10,000 nM	7.7	123.9	0.86	3.3%	n=4
10,000 nM	GST-10 nM	24.2	1,114.3	0.95	1.6%	n=4
	GST-100 nM	26.7	1230.7	0.94	1.8%	n=4
	GST-1000 nM	31.1	1441.0	0.86	4.4%	n=4
	GST-10,000 nM	11.7	515.1	0.87	3.7%	n=4
	FLAG-10,000 nM	6.9	165.3	0.83	4.3%	n=4
